# Epstein-Barr Virus Infection Complicated by a Splenic Infarct in a Patient With Methylenetetrahydrofolate Reductase (MTHFR) Mutation

**DOI:** 10.7759/cureus.79204

**Published:** 2025-02-18

**Authors:** Hassan Fawaz, Mohammad Hassan Hodroj, Nicole Charbel, Firas Kreidieh

**Affiliations:** 1 Internal Medicine, American University of Beirut Medical Center, Beirut, LBN; 2 Hematology-Oncology, American University of Beirut Medical Center, Beirut, LBN

**Keywords:** ebv infection, infectious mononucleosis, mthfr mutation, splenic infarct, therapeutic anticoagulation

## Abstract

Splenic infarction has been reported as a rare complication of infectious mononucleosis (IM), documented in only a few case reports. We present the case of a patient diagnosed with IM complicated by splenic infarction, with thrombophilia workup revealing a homozygous methylenetetrahydrofolate reductase (MTHFR) mutation and elevated homocysteine levels. Infections play a critical role in thrombosis formation through various mechanisms, primarily inflammation due to cytokine production, which alters the coagulation cascade and promotes platelet activation. Elevated homocysteine is considered a weak prothrombotic factor, with its effect amplified by the presence of other risk factors. The prothrombotic effects of homocysteine are poorly understood and are thought to involve proinflammatory effects, oxidative stress, and platelet adhesion. This case adds to the growing body of literature associating Epstein-Barr virus (EBV) infection with splenic infarction. Timely diagnosis and management with anticoagulation therapy can help prevent complications associated with splenic infarction.

## Introduction

Epstein-Barr virus (EBV) is a member of the herpesvirus family and the etiologic agent of heterophile-positive infectious mononucleosis (IM) [1]. IM typically presents with the triad of fever, sore throat, and fatigue, while less common features include pharyngitis, lymphadenopathy, palatal petechiae, periorbital edema, and rash. Although splenomegaly is frequently identified on ultrasonography, it is clinically apparent in only 15%-65% of cases on physical examination [2]. While splenic rupture is a life-threatening complication more commonly reported, splenic infarction remains exceedingly rare. A 2023 systematic review by Toti et al. identified 186 cases of splenic rupture compared to just 29 cases of EBV-associated splenic infarction, with the latter often linked to underlying hematological conditions or thrombophilia [3,4].

Splenic infarction, characterized by ischemic necrosis secondary to compromised vascular perfusion, may result from embolic events, thrombosis, infections, or hematologic disorders [4]. Babesia microti, a malarial parasite, is a recognized infectious trigger of splenic infarction through thrombogenic mechanisms. Infection with B. microti induces hemolytic anemia, endothelial injury via oxidative stress, and microcirculatory obstruction due to cytoadherence of parasitized erythrocytes to vascular walls [5]. These processes collectively potentiate a prothrombotic state, particularly in individuals with genetic or acquired thrombophilia.

Hereditary thrombophilia, such as factor V Leiden (the most common inherited form), predisposes individuals to venous thromboembolism, particularly in atypical sites such as splanchnic or cerebral veins [6]. Less understood is the contribution of methylenetetrahydrofolate reductase (MTHFR) mutations, which impair homocysteine metabolism, leading to hyperhomocysteinemia and associated endothelial dysfunction, oxidative stress, and platelet activation [7]. Elevated homocysteine levels are not exclusive to genetic mutations; they also occur in celiac disease, vitamin B12 deficiency, and chronic inflammatory states, broadening their relevance to thrombosis risk [8]. Despite this, data linking MTHFR mutations to splenic infarction remain scarce, with only isolated cases reported [9,10].

Here, we present the first documented case of IM complicated by splenic infarction in a patient with homozygous MTHFR mutations and marked hyperhomocysteinemia, underscoring the interplay between EBV infection and inherited thrombophilia. We also provide a literature review of EBV-associated splenic infarction, emphasizing the systemic thrombotic risks posed by infection-genetic interactions.

## Case presentation

A 23-year-old male, previously healthy, presented to our institution with a two-week history of progressive sore throat, fever, and fatigue. His physical examination was significant for right tender cervical lymphadenopathy, splenomegaly, and left upper quadrant tenderness.

Laboratory investigations (Table 1) confirmed acute infectious mononucleosis due to EBV, with positive viral capsid antigen (VCA) IgM and IgG antibodies. Mild leukocytosis (white blood cell count: 10,700/μL, lymphocytes: 50%) and significantly elevated inflammatory markers (CRP: 31.1 mg/L) were noted. Liver enzymes were elevated (serum glutamic pyruvic transaminase (SGPT): 179 U/L, serum glutamic-oxaloacetic transaminase (SGOT): 101 U/L), consistent with hepatocellular injury. An abdominal CT scan demonstrated moderate splenomegaly (16 cm craniocaudal length) and prominent mesenteric lymph nodes (largest at 1.5 cm). The patient was discharged with supportive care.

**Table 1:** Laboratory test results before and after splenic infarct. MCV, mean corpuscular volume; SGPT, serum glutamate pyruvate transaminase; SGOT, serum glutamate oxaloacetate transaminase; EBV VCA, Epstein-Barr virus viral capsid antigen; CMV, cytomegalovirus; HBV, hepatitis B virus; HCV, hepatitis C virus; HIV, human immunodeficiency virus

Lab Test	Before Splenic Infarct	After Splenic Infarct	Reference Range
CRP	31.1 mg/L (high)	2.8 mg/L	< 10 mg/L
SGPT (ALT)	179 U/L (high)	21 U/L	7–56 U/L
SGOT (AST)	101 U/L (high)	15 U/L	5–40 U/L
Homocysteine	N/A	35.4 µmol/L (high)	4–15 µmol/L
Folate	N/A	2 ng/mL (low)	> 3 ng/mL
EBV VCA IgM	Positive	Negative	Negative
EBV VCA IgG	Positive	N/A	Negative or past infection
White Blood Cell Count	10,700 /μL	7,000 /μL	4,000–11,000 /μL
Lymphocyte Percentage	50%	38%	—
Platelet Count	175,000 /μL	206,000 /μL	150,000–450,000 /μL
Hemoglobin	16.0 g/dL	15.4 g/dL	13.5–17.5 g/dL (men)
MCV	94.1 fL	89.2 fL	80–100 fL
Creatinine	0.9 mg/dL	N/A	0.6–1.2 mg/dL (men)
Bilirubin Total	0.5 mg/dL	0.4 mg/dL	0.1–1.2 mg/dL
Bilirubin Direct	0.2 mg/dL	0.1 mg/dL	< 0.3 mg/dL
Alkaline Phosphatase	N/A	57 U/L	44–147 U/L
CMV Serology	Negative	N/A	Negative
HBV Serology	Negative	N/A	Negative
HCV Serology	Negative	N/A	Negative
HIV Serology	Negative	N/A	Negative
COVID-19	Negative	N/A	Negative

**Figure 1:**
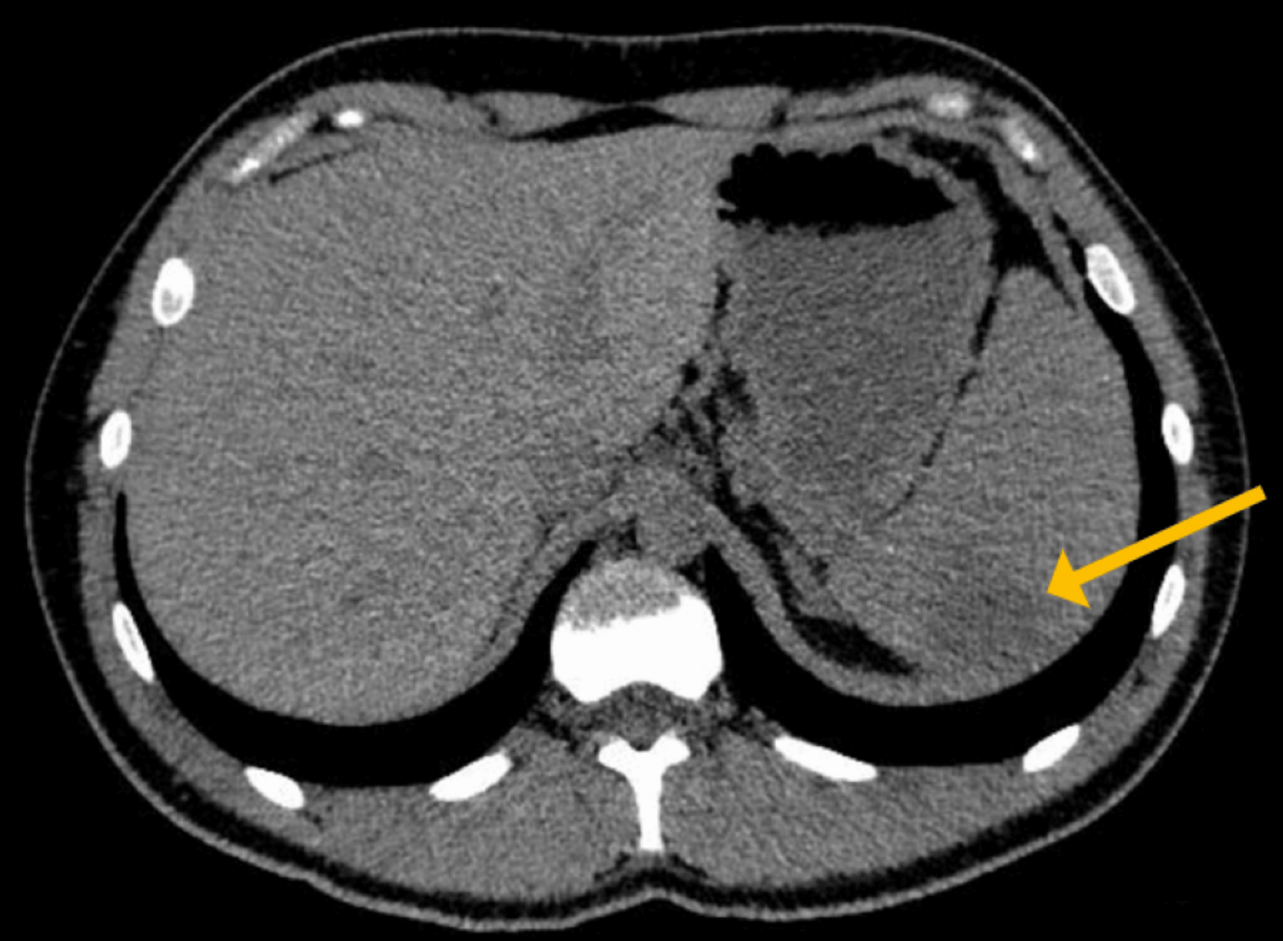
Abdominal CT scan without contrast showing splenomegaly (15.6 cm in craniocaudal dimensions) with a hypodensity at the posterior part of the upper pole of the spleen measuring 4 x 3 cm, corresponding to a splenic infarct.

Two months later, he returned with acute-onset severe left upper quadrant pain and worsening fatigue. A repeat CT scan revealed partial resolution of lymphadenopathy, persistent splenomegaly (15.6 cm craniocaudal length), and a new wedge-shaped splenic infarct measuring 4 x 3 cm (Figure 1).

Thrombophilia workup showed normal partial thromboplastin time (PTT), international normalized ratio (INR), D-dimer, fibrinogen, antithrombin III, and protein C and S activity. Tests for anticardiolipin antibodies, lupus anticoagulant, and JAK2 mutation were negative. Homocysteine levels, however, were markedly elevated at 35.4 μmol/L, and genetic analysis confirmed a homozygous MTHFR C677T mutation. Factor V Leiden and factor II (G20210A) mutations were not detected. Low folate levels (2 ng/mL) with normal vitamin B12 suggested a nutritional contribution to hyperhomocysteinemia.

The elevated homocysteine level, likely driven by the MTHFR mutation, was linked to the patient’s splenic infarction, highlighting a prothrombotic state. He was initially treated with enoxaparin (80 mg subcutaneously twice daily for one week) and subsequently switched to rivaroxaban (15 mg twice daily for two weeks, then 20 mg daily), along with folic acid and vitamin B12 supplementation.

Two months later, a follow-up abdominal CT scan showed complete resolution of the splenic infarction and normalization of homocysteine levels.

A comprehensive differential diagnosis was performed. Embolic sources, such as atrial fibrillation and endocarditis, were excluded based on a normal cardiac evaluation. Hematologic disorders, including JAK2-positive myeloproliferative neoplasms, were ruled out. Tests for antiphospholipid syndrome and common thrombophilias, including factor V Leiden and prothrombin mutations, were negative, leaving hyperhomocysteinemia as the only identified contributor.

The hyperhomocysteinemia was attributed to the homozygous MTHFR C677T mutation present in this patient. Rare conditions such as cystathionine beta-synthase (CBS) deficiency were excluded clinically. Nutritional deficiencies were also considered likely contributors, given the rapid response to folate and vitamin B12 supplementation. Renal causes were ruled out based on normal kidney function.

## Discussion

Splenic infarction is a rare but serious complication of IM. A systematic review by Toti et al. identified 29 cases of EBV-associated splenic infarction, typically occurring in young, otherwise healthy individuals within two to four weeks of infection onset. The proposed mechanism involves virus-induced splenomegaly and capsular stretching, leading to vascular compromise [3]. However, in our patient, splenic infarction developed two months after the initial EBV diagnosis, suggesting a distinct mechanism driven by thrombophilia rather than acute mechanical stress. Notably, the review found that only 21% of cases had preexisting hematologic disease, highlighting the potential underdiagnosis of hypercoagulable states in delayed infarction presentations.

While EBV-related thrombosis most commonly affects the spleen due to localized vascular congestion, systemic thrombotic events, such as deep vein thrombosis, have been reported in individuals with coexisting prothrombotic factors [11]. In our case, the homozygous MTHFR C677T mutation led to severe hyperhomocysteinemia, which, in synergy with EBV-driven inflammation, likely precipitated splenic infarction. This aligns with Toti et al.’s observation that delayed splenic complications frequently coincide with underlying hypercoagulable disorders [3].

EBV induces a prothrombotic state through cytokine-mediated endothelial activation and tissue factor upregulation, promoting vascular inflammation and coagulation [12]. Hyperhomocysteinemia further amplifies these effects by increasing endothelial oxidative stress, impairing nitric oxide bioavailability, and enhancing platelet adhesion [13]. In our case, the timeline of EBV infection, followed by progressive homocysteine elevation and eventual infarction, strongly suggests a causal relationship. The absence of other thrombophilias (normal antiphospholipid antibodies, protein C/S, factor V Leiden, and JAK2 mutations) further isolates MTHFR-related hyperhomocysteinemia as the primary contributor.

Other infections, such as B. microti, have also been implicated in splenic infarction. Babesia, a tick-borne parasite, induces hemolysis and splenic sequestration, leading to microvascular occlusion [5]. In contrast, EBV promotes thrombosis indirectly by exacerbating inflammatory and procoagulant pathways in individuals with preexisting hypercoagulable conditions [3].

Few cases of splenic infarction have been reported in patients with a similar profile of low folate, elevated homocysteine, and an MTHFR mutation [9,10,14]. The last two factors are more commonly associated with VTE [6,7]. However, the role of MTHFR mutation and hyperhomocysteinemia in VTE remains controversial; while some studies have reported a significant association [15,16], others have not found a clear link [17,18].

This case highlights the rare occurrence of splenic infarction as a complication of IM, underscores the importance of thrombophilia evaluation in patients presenting with delayed infarction, and adds to the growing body of literature associating EBV infection with thrombotic complications.

## Conclusions

This case highlights a rare, delayed splenic infarction, occurring two months after EBV infection in a patient with a homozygous MTHFR mutation and hyperhomocysteinemia. While the prothrombotic significance of MTHFR mutations and elevated homocysteine remains controversial, EBV-induced inflammation may have exacerbated their thrombogenic potential, particularly in the absence of other thrombophilias. Early diagnosis and appropriate anticoagulation therapy can prevent complications and improve outcomes. Further studies are needed to better define the role of genetic and metabolic factors in EBV-associated thrombotic events.
